# Evaluating the Antioxidant and Antidiabetic Properties of *Medicago sativa* and *Solidago virgaurea* Polyphenolic-Rich Extracts

**DOI:** 10.3390/molecules29020326

**Published:** 2024-01-09

**Authors:** Gabriela Paun, Elena Neagu, Andreia Alecu, Camelia Albu, Ana-Maria Seciu-Grama, Gabriel Lucian Radu

**Affiliations:** National Institute for Research-Development of Biological Sciences, Centre of Bioanalysis, 296 Spl. Independentei, P.O. Box 17-16, 060031 Bucharest, Romania; andreia.alecu@incdsb.ro (A.A.); camelia.albu@incdsb.ro (C.A.); anamaria.seciu@incdsb.ro (A.-M.S.-G.); lucian.radu@incdsb.ro (G.L.R.)

**Keywords:** green extraction, antioxidant, antidiabetic, *Medicago sativa*, *Solidago virgaurea*, polyphenolic-rich extract

## Abstract

The present study evaluated the antioxidant and antidiabetic properties of *Medicago sativa* and *Solidago virgaurea* extracts enriched in polyphenolic compounds. The extracts were obtained by accelerated solvent extraction (ASE) and laser irradiation. Then, microfiltration was used for purification, followed by nanofiltration used to concentrate the two extracts. The obtained extracts were analyzed to determine their antioxidant activity using DPPH radical scavenging and reducing power methods. The antidiabetic properties have been investigated in vitro on a murine insulinoma cell line (β-TC-6) by the inhibition of α-amylase and α-glucosidase. *M. sativa* obtained by laser irradiation and concentrated by nanofiltration showed the highest DPPH• scavenging (EC_50_ = 105.2 ± 1.1 µg/mL) and reducing power activities (EC_50_ = 40.98 ± 0.2 µg/mL). *M. sativa* extracts had higher inhibition on α-amylase (IC_50_ = 23.9 ± 1.2 µg/mL for concentrated extract obtained after ASE, and 26.8 ± 1.1), while *S. virgaurea* had the highest α-glucosidase inhibition (9.3 ± 0.9 µg/mL for concentrated extract obtained after ASE, and 8.6 ± 0.7 µg/mL for concentrated extract obtained after laser extraction). The obtained results after evaluating in vitro the antidiabetic activity showed that the treatment with *M. sativa* and *S. virgaurea* polyphenolic-rich extracts stimulated the insulin secretion of β-TC-6 cells, both under normal conditions and under hyperglycemic conditions as well. This paper argues that *M. sativa* and *S. virgaurea* polyphenolic-rich extracts could be excellent natural sources with promising antidiabetic potential.

## 1. Introduction

Type 2 diabetes mellitus (DM) is a major public health problem due to its continuously increasing prevalence, chronic complications, and because it is a major risk factor for cardiovascular diseases with economic and social implications. Prior studies have shown that synthetic antidiabetic drugs used for treating type 2 diabetes are associated with adverse side effects. For instance, thiazolidinedione causes heart failure, and sulfonylureas causes hypoglycemia and weight gain [1]. The interest in natural biologically active compounds comes from the recognition that they have very few side effects compared to synthetic compounds and that research in recent decades demonstrated the importance of the synergistic effect of bioactive compounds in a natural mixture. The high cost of synthetic antidiabetic drugs and adverse side effects make it necessary to seek a green and cheap alternative solution to managing this disease.

Natural products have recently gained great interest for diabetic treatment, including traditional herbal remedies, plant extracts, and their chemical components [2]. Polyphenols (phenolic acids, flavones, and isoflavones) are compounds of widespread interest in the nutritional and medicinal fields. These natural compounds are considered essential components due to their antioxidative, anti-inflammatory, antimicrobial, anti-mutagenic, estrogenic, and anticancer properties, combined with their ability to modulate critical cellular enzyme functions [3,4,5].

The interest in natural biologically active compounds comes from the recognition that they have very few side effects compared to synthetic compounds, and from recently conducted research, which demonstrated the importance of the synergistic effect of bioactive compounds in a natural mixture. Some studies reported that there is a relationship between the antidiabetic and antioxidant properties of medicinal plants [6]. A therapeutic alternative for diabetes treatment consists of the reduction in post-prandial hyperglycemia with natural products. This is obtained by delaying glucose absorption via α-glucosidase and α-amylase inhibiting, enzymes involved in carbohydrate hydrolysis in the digestive tract [7]. Many studies have highlighted that bioactive compounds with antioxidant activity, especially polyphenols, have the ability to inhibit these enzymes and could be beneficial in the management of diabetes mellitus [2,8].

Studies showed that a good choice of extraction technique, solvents, and extraction conditions might support the content of targeted bioactive compounds in the final extract, leading to increased efficacy of the final product. Although intensive investigations have been conducted in recent decades, researchers are still seeking stable plant sources of natural antioxidants and highly efficient extraction technologies. Nowadays, there is more interest in green and sustainable extraction methods [9,10,11,12]. Green methods offer the advantages of a shorter extraction time, higher selectivity, and lower organic solvent expense. Lately, ultrasound-assisted and accelerated solvent extraction (ASE), using organic solvents at high pressure and temperature, has been intensively studied [13,14,15,16]. Recently, a new method called laser irradiation showed great value in extractive technology. Yet there is only one study related to this method [17]. Laser irradiation is used to intensify the exposed environment’s heating and increase the reaction speed of the extraction process, resulting in an increased amount of biologically active substances extracted from plants (e.g., anthocyanins, polysaccharides, proteins, polyphenolics, minerals, etc.) [18,19].

*Medicago sativa* (lucerne; Fabaceae family) contains significant quantities of polyphenols, especially isoflavones known as phytoestrogens [20,21]. *M. sativa* is one of the most prevalent forage crops, but it also has a long tradition of use in folk medicine for central nervous and digestive system disorders and for curing other ailments, including cancer [22,23,24,25]. However, only a few research studies have been directed toward the antidiabetic potential of *M. sativa* [26,27,28].

*Solidago virgaurea* (goldenrod; Asteraceae family) is a medicinal plant used in popular medicine to treat numerous diseases, especially as a urological agent in kidney and bladder inflammation [29,30]. A recent study showed the antidiabetic potentials of this plant [31]. According to the literature, its pharmacodynamic activity is attributed to the presence of biologically active compounds, especially flavonoids, considered to be the most essential [20,32,33].

Since flavonoids are thermolabile compounds, high consideration was paid to the extraction and concentration of these compounds in the present study. In this context, we compared the antioxidant and antidiabetic activities of the two medicinal plant extracts enriched in polyphenols, using accelerated solvent extraction and laser irradiation extraction, coupled with concentration by nanofiltration. These obtained final products are meant to be used in antidiabetic management from safe and natural resources. The in vitro antidiabetic study was carried out by inhibiting amylase and glucosidase, as well as on a murine insulinoma cell line (β-TC-6).

## 2. Results and Discussion

### 2.1. Chemical Characterization

The first objective of the study was to obtain the polyphenolic-rich extracts of *Medicago sativa* and *Solidago virgaurea* using two green extraction methods: accelerated solvent extraction (ASE) and laser irradiation extraction (LE), followed by nanofiltration, used to concentrate the extracts. The previous results of the authors demonstrated the efficiency of the nanofiltration process in the concentration of polyphenolic compounds (phenolic acids, flavonoids, and isoflavonoids) [34].

Accelerated solvent extraction (ASE) involves the use of solvents at high temperatures and pressures. High temperatures accelerate the kinetics of the extraction process, while increased pressure keeps the solvent below its boiling point, thus obtaining fast and safe extractions. Some parameters such as solvent, temperature, the number of extraction cycles, and the static time are very important for the efficient extraction of polyphenolic compounds. However, due to the particularities of the compounds of interest and the fact that, at high temperatures, their structure could be damaged, leading to loss of their activity, the extraction was carried out in 3 extraction cycles performed at 60 °C [35]. Another study showed that the optimal ASE parameters were 50% ethanol, 150 °C, two extraction cycles, and 10 min static time [36].

The HPLC-MS method has been used for the phenolic acid, flavonoid, and isoflavonoid profile characterization of plant extract samples. The target bioactive compounds are presented in Table 1.

The data obtained showed that laser irradiation is an efficient extraction method for some flavonoids and isoflavonoids (e.g., quercetin 3-D glucoside, quercitrin, naringenin, and vitexin), but both methods ensure efficient extraction of the desired compounds. Hence, this extraction method enhancing these valuable compounds is of particular interest, especially because it has been poorly studied thus far. Laser irradiation is a very new method of selective extraction, which demonstrated the efficiency in the extraction of polyphenols from plants at 552 nm, 660 nm, and 785 nm [17]. This method was effective in the case of flavonoid and isoflavonoid compound extraction from our studied plants, leading to a significant amount of extract (depending on the capacity of the extractor) obtained within less time.

However, this is the first study that uses this combination of wavelengths in laser extraction and that demonstrates the efficiency in the extraction, especially of flavonoids and isoflavonoids.

At the same time, with ASE, higher values were obtained for other compounds from the class of flavonoids and isoflavonoids. The comparison of the total flavonoid and isoflavonoid compound values in the samples indicates close values for the extracts obtained by both methods. Using a 50% (*v*/*v*) hydroalcoholic solution represents a reduction in the cost of the extraction process versus using pure solvents while maintaining a high extraction yield of the targeted compounds. This could be explained due to the different solubility of the extracted polyphenolic compounds, some of which have a higher solubility in water (phenolic acids), while others are extracted with a higher yield in ethanol.

Several studies indicated that *M. sativa* is a rich source of phytoestrogens. HPLC-MS analysis showed that the highest rutin content was the dominant flavonoid in all of the plant extracts. Rutin, quercetin, kaempferol, naringenin, formononetin, and genistein were also reported in other studies [18,20]. Tucak et al. found that genistein represents 30.33% of total phytoestrogens, followed by kaempferol, with 26.84% in the total amount of phytoestrogens [37]. However, biochanin A and vitexin were detected only in *M. sativa* seeds and sprouts, not in the aerial part [38]. Among the isoflavones, vitexin is found in the largest amount. The determined values of the phytoestrogens investigated in this research differed from the results obtained by the above-mentioned studies, which are most likely related to the type of cultivar, stages of maturity, extraction method, and other factors. Chlorogenic acid was the main phenolic acid from all extracts.

The HPLC–MS analysis of *S. virgaurea* extracts showed a significant content of rutin, quercetin 3-β-D-glucoside, and vitexin. Daidzein was not detected in *S. virgaurea* extracts. Our data confirmed the results of previously published studies reporting significant amounts of rutin in *S. virgaurea* species [32]. To the best of our knowledge, formononetin, biochanin A, and vitexin were not reported previously in goldenrod hydroalcoholic extracts.

### 2.2. Total Antioxidant Activity

Polyphenolic compounds are a group of natural compounds with a biologically active potential; hence, they have an antioxidant effect. The results for the total flavonoid content and antioxidant activity (DPPH and Fe(III) reducing power methods) in the various extracts compared with ascorbic acid (vitamin C) as standard, known for its antioxidant properties, are displayed in Table 2. 

The DPPH scavenging assay is a frequently utilized method to evaluate antioxidant activity. The EC_50_ values related to the DPPH radical scavenging activity for all extracts were higher than vitamin C, showing a moderate antioxidant activity, with *M. sativa* obtained by laser irradiation and concentrated by nanofiltration being the most active extract (EC_50_ = 105.2 ± 1.1 µg/mL). The free radical inhibition results for *M. sativa* are in accord with the previous study, which showed strong antioxidant activity of extracts obtained from *M. sativa* (IC_50_ of the extract = 245.18 ± 48.41 μg/mL) [39].

Comparing the obtained results, we can observe that although the extracts of *S. virgaurea* have a much higher content of flavonoids, they have a lower antioxidant activity than *M. sativa*. This result can be explained by a higher content of other compounds, with the antioxidant activity present in the extracts of *M. sativa*, such as phenolic acids or other isoflavone compounds, not quantified in the studied extracts. Our results about the antioxidant activity of *S. virgaurea* extracts confirm the results of the other research, but it must be taken into account that the antioxidant activity is dependent on the solvent and the extraction method applied [40].

The reducing power showed significant differences between the examined extracts compared to ascorbic acid as a standard, due to the highest EC_50_ value obtained. *M. sativa* polyphenolic-rich extracts had the highest reducing power activity. The reducing power of the extracts can be determined by the hydrogen donation ability, which stabilizes the molecules by acceptance of hydrogen ions in the extracts. The reducing power results revealed that all tested extracts had good abilities to donate electrons, which were involved in the antioxidant activity.

The DPPH assay can be applied to both lipophilic and hydrophilic compounds, while the reducing power assay was more sensitive to hydrophilic compounds [41].

The free radical scavenging activity and the reducing power depend on the number of hydroxyl groups attached to a benzene ring, as such groups could donate hydrogen to stabilize free radicals. Thus, the lack of a positive correlation between the reducing power and the scavenging DPPH free radical activity may only result from different reaction mechanisms and steric accessibilities of the reagents and each type of antioxidant in the two assays.

### 2.3. Antidiabetic Activity

- α-amylase and α-glucosidase inhibition

One of the alternative approaches regarding the prevention/modulation of postprandial hyperglycemia consists of using natural therapeutic inhibitors of α-amylase and α-glucosidase, as they are key enzymes in starch digestion. Our results for these enzymes’ inhibition by the tested polyphenolic-rich extracts are presented in Table 3.

*M. sativa* extracts had higher inhibitory activity on α-amylase (IC_50_ = 23.9 ± 1.2 µg/mL for concentrated extract obtained after ASE, and 26.8 ± 1.1 µg/mL for concentrated extract obtained after LE), while *S. virgaurea* had the highest α-glucosidase inhibition compared with acarbose, used as standard. *S. virgaurea* extracts showed the best α-glucosidase inhibition (IC_50_ of 9.3 ± 0.9 µg/mL for concentrated extract obtained after ASE, and 8.6 ± 0.7 µg/mL for concentrated extract obtained after LE), almost 7 times lower than acarbose (IC_50_ of 66.5 ± 4.2 µg/mL).

The inhibitory activities of the rutin, the main compound identified in extracts, were higher than those of the acarbose. Thus, rutin can be considered one of the compounds responsible for the activity of the extracts. The milder inhibition of α-amylase than α-glucosidase of all studied extracts could eliminate the major drawback of current drugs with side effects [42].

Flavonoids, such as rutin, quercitrin, and isoquercitrin (quercetin 3-β-D-glucoside), but also isoflavones (daidzein, genistein, vitexin), have been previously reported to have a hypoglycemic effect and stronger inhibitory effect on α-glucosidase. [43,44,45]. In recent years, several studies have reported the inhibitory activity of phenolic acids on α-amylase and α-glucosidase. Latest studies showed that the interaction between polyphenolic compounds and α-amylase/α-glucosidase consists of the hydrogen bond between the amino acid residues of the enzymes and the hydroxyl groups in the polyphenolic compounds. These interactions disrupt the protein structure of the enzymes, leading to a decrease in enzyme activity [46].

The polyphenols’ inhibitory activity on α-amylase and α-glucosidase has been related to the number of hydroxyl groups, the number of double bonds on aromatic rings A and B, as well as the heterocyclic ring C [47]. Thus, the phenolic acids with more hydroxyl groups (chlorogenic, caffeic, and gallic acids) presented a higher inhibition effect against both enzymes [48].

This study suggests that combinations of polyphenolic compounds from the studied plants have a synergic effect on α-amylase and α-glucosidase inhibition.

Recent in vivo studies showed the anti-hyperglycemic effect of *S. virgaurea*, but the antidiabetic activity of *S. virgaurea* has rarely been studied [49]. However, the α-amylase and α-glucosidase inhibition by *S. virgaurea* extract was not found in the literature.

- Effect of extracts on insulin secretion by β-TC6 cell lines

In this study, we investigated the complementary in vitro antidiabetic activity of *M. sativa* and *S. virgaurea* polyphenolic-rich extracts, at the tested concentrations (10–250 μg/mL), on a murine insulinoma cell line (β-TC-6).

**Figure 1:**
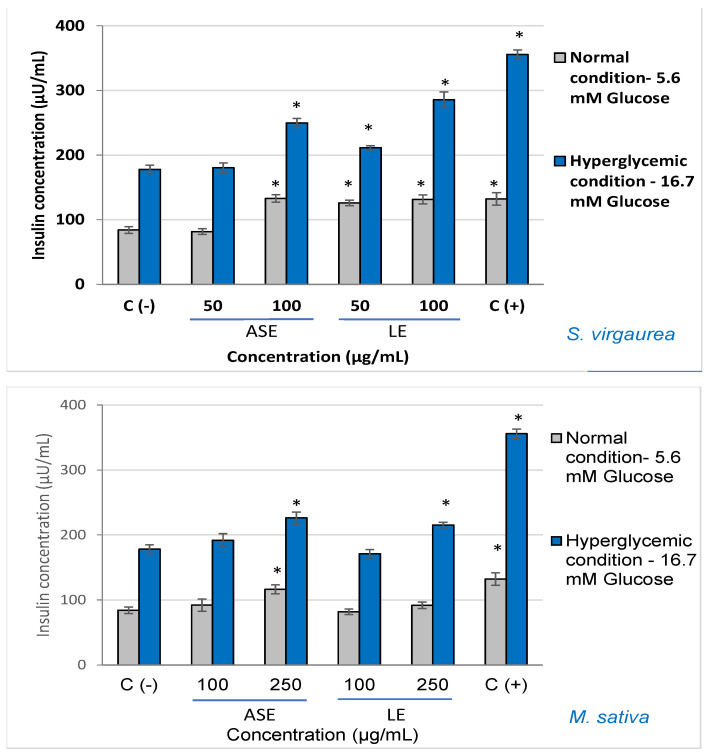
Effect of polyphenolic-rich extracts on insulin secretion, after treatment with different concentrations, in both normal and hyperglycemic conditions. C (−)—negative control, untreated cells. C (+)—positive control, cells treated with alanine. * *p* < 0.05, compared to the untreated cells.

The obtained results showed that higher insulin concentrations were obtained after treatment with all the tested extracts compared to the control, both in normal conditions (5.6 mM) and in hyperglycemic conditions (16.7 mM) (Figure 1).

Under normal glycemic conditions (5.6 mM), the highest concentration of the insulin was found in two specific cases of cell treatment with either 50 µg/mL (~134 µU/mL) *S. virgaurea* concentrated extract obtained by ASE extraction or cell treatment with 100 µg/mL (~132 µU/mL) *S. virgaurea* concentrated extract obtained by LE extraction. In the case of the control stimulated with 5.6 mM glucose and untreated, the secreted insulin concentration was ~84 μU/mL.

Likewise, similar results of the effectiveness of stimulating insulin secretion were also obtained in hyperglycemic conditions (16.7 mM). Thus, the best results were obtained after the treatment with *S. virgaurea* concentrated extract obtained after ASE extraction at 50 µg/mL (~249 µU/mL), *S. virgaurea* concentrated extract obtained after LE extraction at 100 µg/mL (~286 µU/mL), and *M. sativa* extracts 250 µg/mL; for the stimulated and untreated control, the secreted insulin concentration was ~178 μU/mL. Alanine was used as a positive control, demonstrating its ability to significantly stimulate insulin secretion.

Interestingly, even though *M. sativa*, a related and well-known phytotherapeutic plant, is traditionally used as an anti-diabetic agent, its α-glucosidase- and α-amylase-inhibitory properties were poorly investigated [50].

## 3. Materials and Methods

### 3.1. Materials

Flavonoid and isoflavonoid compounds, including rutin, quercitrin, quercetin 3-β-D-glucoside, quercetin, isorhamnetin, formononetin, genistein, naringenin, biochanin A, and vitexin, were purchased from Sigma–Aldrich (Schnelldorf, Germany), daidzein was obtained from Fluka (Buchs, Switzerland), luteolin and kaempferol were purchased from Carl Roth (Karlsruhe, Germany), 2,2-difenil-1-picrilhidrazil (DPPH), potassium ferricyanide, sodium carbonate (Na_2_CO_3_), dinitrosalicylic acid (DNS), α-amylase from hog pancreas, α-glucosidase from *Saccharomyces cerevisiae*, and 4-nitrophenyl α-d-glucopyranoside (NPG) were purchased from Sigma–Aldrich, and iron chloride was bought from Fluka. All other used reagents, methanol (Riedel-de Haen), and ethanol (Chemical Company) were of chromatographic or analytical purity; the ultra-pure water was obtained using the distillation apparatus from Evoqua Water Technologies (Pittsburgh, PA, USA).

The medicinal plants were collected from the Cluj county (Romania) natural site, and voucher specimens were stored in the Herbarium of Babes-Bolyai University from Cluj-Napoca (code: 868.786 for *Solidago virgaurea* L.; code: 622172 for *Medicago sativa*).

### 3.2. Extract Preparation

Two green extraction methods were used to study their influence on bioactive compound extraction: accelerated solvent extraction (ASE) and laser irradiation (LE).

#### 3.2.1. ASE Extraction

Accelerate solvent extraction of dried and grounded *M. sativa* and *S. virgaurea* was realized by Dionex ASE 350 System (Thermo Fisher Scientific Inc., Waltham, MA USA). Each stainless steel cell (100 mL) equipped with a cellulose filter was filled with 15 g of dried and fine-milled plant and diatomaceous earth and the ASE conditions were set as solvent—ethanol/water (50/50, *v*/*v*), temperature—60 °C, static time—10 min, number of cycles—3. The extracts were collected in a 250 mL flask and stored at 4 °C before concentration. According to the ASE extract volume, the concentration of the extracts was 9% (*w*/*v*).

#### 3.2.2. Laser Irradiation Extraction

**Figure 2:**
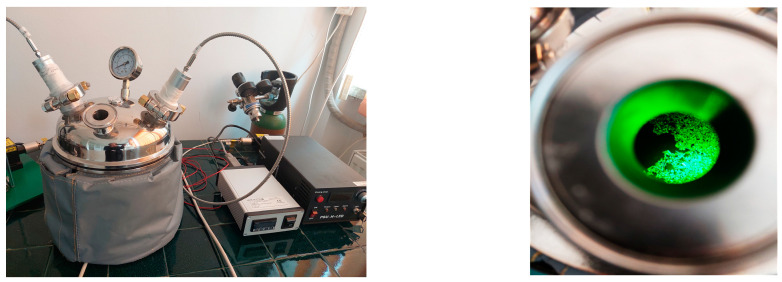
Laser-assisted extraction installation.

LE extraction was performed in the same conditions with ASE; 9 g of the dry plant (aerial parts) was mixed with 100 mL of ethanol/water (50/50, *v*/*v*), and extracted for 30 min assisted with laser radiation at a combined 1270 and 1550 nm. The LE extraction used a steel extractor (made by the Apel Laser S.R.L) provided with a lid with two windows through which laser irradiation was carried out (Figure 2).

Subsequently, all extracts were purified by microfiltration through a Millipore membrane (0.2 µm pores) and concentrated by nanofiltration through Sterlitech membranes NF90 with a cut-off of 150–300 Da using a KMS Laboratory Cell CF-1 module. The concentrated extracts were stored in a freezer at −20 °C for use in further analysis.

### 3.3. Analysis of Polyphenolic Compounds

#### 3.3.1. Quantification of Total Polyphenols and Flavonoids

Polyphenolic compound content determination was performed using the Folin–Ciocalteu method [51]. A total of 2 mL extract and 2 mL Folin–Ciocalteu reagent were mixed and filtered; then, 0.5 mL of the filtrate was added to 9.5 mL sodium carbonate 20% and absorbance was read at 760 nm. A calibration curve of different concentrations of chlorogenic acid solutions (10 µg/mL–1000 µg/mL) was used for expressing the results as chlorogenic acid equivalent (CA), with the equation y = 0.0016x + 0.013 (R^2^ = 0.9945).

Total flavonoid content was quantified using the aluminum chloride colorimetric method [52]. A total of 2 mL of extract and 3 mL of methanol were mixed. After filtration, 1 mL filtrate was added to 1 mL sodium acetate solution, 0.6 mL of aluminum chloride solution, and 2.4 mL methanol. The absorbance was measured at 430 nm and the flavonoid content was calculated based on a rutin calibration curve (y = 0.0073x − 0.0357; R^2^ = 0.9959).

#### 3.3.2. HPLC-MS Analysis

HPLC analysis was realized using an HPLC Shimadzu system consisting of a SIL-20AC autosampler, two LC-20AD pumps, a DGU-20A degasser, and a CTO-20A column oven with an LC Solution software var. 5.1. The HPLC was coupled to a mass spectrometer detector, LCMS-2010 with an ESI interface using negative ionization mode using the following parameters: detector voltage, 1.8 kV; interface voltage 4 kV; heat block temperature, 200 °C; CDL temperature, 200 °C; interface temperature, 250 °C; and nebulization gas (N_2_) flow rate, 1.5 L min^−1^. A previously developed HPLC-MS method [53] was used for the identification and quantification of polyphenol compounds, and analyses were performed on a Kromasil 100-5-C18 2.1 × 150 mm column and with an elution gradient of mobile phase (formic acid in water, pH = 3, solvent A and formic acid in MeCN, pH = 3, solvent B, 0–20 min 5–30% solvent B, 20.01–40 min 30% solvent B, 40.01–50 min 50% solvent B, 50.01–52 min 50–5% solvent B, 52.01–62 min 5% solvent B) and a gradient of flow rate (0.1 mL min^−1^ from 0–5, 15.01–35, and 60.01–62 min and 0.2 mL min^−1^ between 5.01–15 and 35.01–60 min). The selected ion monitoring (SIM) mode was used and the corresponding peaks of the compound fragment ions ([M-H]^-^: 163, 169, 179, 253, 267, 269, 271, 283, 285, 301, 315, 317 353, 431, 447, 463, and 609) were obtained for quantitative analysis.

### 3.4. Antioxidant Assays

#### 3.4.1. DPPH Radical Scavenging

The DPPH assay was carried out as described by Bondet et al. [54] with slight modifications. A total of 100 µL extract with different concentrations was mixed with 1000 µL DPPH (2,2-diphenyl-1-picrylhydrazyl), 0.25 mM solution, and 1.9 mL methanol. The absorbance was measured at 517 nm, and the extracts’ scavenging activity was determined by the following formula:

RSA (%) = [(Ac − As)/Ac] × 100,

where RSA = radical scavenging activity; Ac = control absorbance; and As = sample absorbance. Results were presented as inhibition, in EC_50_ (μg/mL).

#### 3.4.2. Fe (III) Reducing Power Assay

The reducing power assay is based on the reduction of iron (III) to iron (II) and was performed using Berker’s method [55]. The polyphenolic-rich extracts (0.1 mL with varying concentrations) were mixed with 2.5 mL sodium phosphate buffer (0.2 M) and 2.5 mL potassium ferricyanide (1%) and then were kept at 50 °C for 20 min. Thereafter, 2.5 mL of trichloroacetic acid (10%) was added. Finally, an aliquot of 2.5 mL mixture was combined with 2.5 mL water followed by 0.5 mL of iron chloride solution (0.1%) and UV absorbance was read at 700 nm. Results were presented as EC_50_ (μg/mL), the extract concentration giving an absorbance of 0.5 for reducing power, and was calculated from the graph of absorbance against extract concentration.

### 3.5. Antidiabetic Assay

#### 3.5.1. α-Amylase- and α-Glucosidase-Inhibitory Activities

The ability of extracts to inhibit α-amylase and α-glycosidase enzymes was examined to establish the plant’s potential as an antidiabetic.

The α-amylase inhibition analysis was achieved according to our previous study [56]. Briefly, 100 μL of the extracts was added to 250 μL α-amylase from hog pancreas (EC 3.2.1.1) solution in phosphate buffer (pH 6.9) and was maintained at 37 °C for 20 min. Then, 250 μL starch solution was added and incubated at 37 °C for 30 min. Subsequently, 500 μL DNS was added, and the mixture was heated at 90 °C for 5 min. Absorbance measurements were performed at 540 nm.

The α-glucosidase-inhibitory activity was evaluated using a slightly modified method of Ranilla et al. [57]. Samples (60 μL) with different concentrations were incubated with 120 μL of α-glucosidase from *Saccharomyces cerevisiae* (EC 3.2.1.20) solution (0.5 U/mL) and 720 μL phosphate buffer (0.1 M, pH 6.9), at 37 °C, for 15 min. After that, 120 μL of NPG substrate solution was added, and the mixture was incubated at 37 °C, for 15 min. Then, 480 μL of 0.2 M Na_2_CO_3_ solution was added to this mixture to stop the reaction, and the absorbance was read at 405 nm. The results were calculated using the following formula:

$$\%\;\mathrm{enzyme}\;\mathrm{inhibition}=\frac{\Delta A_{control}-\Delta A_{sample}}{\Delta A_{control}}\times 100$$

Values were compared with the standard drug acarbose. IC_50_ values (concentration of the extract that inhibits 50% enzyme activity) were obtained from the linear regression analysis.

#### 3.5.2. In Vitro Insulin Secretion Assay

In vitro cytocompatibility of extracts was evaluated on pancreatic βTC-6 cells in an experimental model of direct contact by MTT assay. The results obtained showed that extracts were biocompatible in the range of 10–100 µg/mL for *S. virgaurea* extracts and 10–250 µg/mL for *M. sativa* extracts. At higher concentrations, cellular viability decreased in a dose-dependent manner. Based on the results obtained by MTT assay, we selected the highest concentrations at which samples did not affect cell viability (>80%) and we tested them further to detect the extracts’ effect on insulin secretion in an in vitro model.

MTT assay was used for cytocompatibility testing according to the international standard ISO 10993-5/2009, as previously described [58].

In vitro evaluation of antidiabetic activity was conducted on a mice insulinoma cell line (βTC-6). βTC-6 cells were purchased from Cell Lines Service (CLS, Germany) and were grown in DMEM medium supplemented with 10% FBS (fetal bovine serum) and 1% PSN (penicillin−streptomycin–neomycin) antibiotic mixture at 37 °C and 5% CO_2_. βTC-6 cells were seeded in a 24-well plate at a density of 1 × 10^5^ cells/mL. After 24 h of cultivation in standard conditions, βTC-6 cells were cultivated in normal (5.6 mM) and hyperglycemic (16.7 mM) conditions in the absence and presence of extracts for 1 h, at 37 °C. The culture medium was then collected, centrifuged for 10 min at 1500 rpm, and stored at −20 °C until insulin measurement. Insulin secretion was determined by Mouse Ins1/Insulin-1 ELISA Kit, according to the manufacturer’s recommendations (Sigma–Aldrich Chemie GmbH, Schnelldorf, Germany). L-alanine (10 mM) was used as the reference stimulant of insulin secretion from pancreatic beta cells. Alanine was used as a positive control because it has been noted to stimulate insulin secretion significantly. Studies have shown that alanine is consumed by b-cell lines and islet cells and increases insulin secretion, findings that are supported by observations from other β-cell lines, including murine and human [59].

### 3.6. Statistical Analysis

Three independent experiments were carried out and the obtained data were presented as mean ± standard deviation (SD) (*n* = 3). The sample pair of interest was analyzed using the paired Student’s *t*-test (Microsoft Excel 2018 software). Significant statistical differences were considered *p* < 0.05.

## 4. Conclusions

In this research, *Medicago sativa* and *Solidago virgaurea* extracts were obtained using accelerated solvent extraction and laser irradiation extraction, coupled with concentration by nanofiltration. The laser irradiation method at the combined wavelengths of 1270 and 1550 nm, reported for the first time in this paper, was efficient in the case of flavonoid and isoflavonoid compound extraction from *M. sativa* and *S. virgaurea*. *M. sativa* polyphenolic-rich extracts had the best values for antioxidant activity (DPPH and Fe(III) reducing power methods). The polyphenolic-rich extracts from both plants showed a significative inhibition on α-amylase and α-glucosidase, correlated with their total phenolic acid, flavonoid, and isoflavonoid high contents. Additionally, the obtained results showed that the studied extracts stimulate insulin secretion in vitro. *S. virgaurea* polyphenolic-rich extracts showed the strongest stimulatory effect on insulin secretion in the in vitro β-TC-6 pancreatic beta cell stimulation model. Our results revealed that the *M. sativa* and *S. virgaurea* enriched with polyphenols could be used as an alternative therapy in the management of diabetes.

Further studies should be designed to explore the mechanism of inhibition of digestive enzymes by the studied extracts.

## Figures and Tables

**Table 1:** Contents of target compounds in the extracts.

Compound	*M. sativa* Polyphenolic-Rich Extract (µg/mL)		*S. virgaurea* Polyphenolic-Rich Extract (μg/mL)	
	Conc. ASE	Conc. LE	Conc. ASE	Conc. LE
Coumaric acid	2.71 ± 0.1	6.18 ± 0.4	7.79 ± 0.6	4.84 ± 0.3
Gallic acid	9.15 ± 0.4	10.27 ± 0.6	16.9 ± 1.2	35.31 ± 2.6
Caffeic acid	1.1 ± 0.05	0.7 ± 0.05	41.24 ± 3.6	25.54 ± 1.4
Ellagic acid	1.66 ± 0.08	4.25 ± 0.2	10.6 ± 0.9	9.57 ± 0.8
Chlorogenic acid	263.81 ± 11.2	112.58 ± 8.2	2343.78 ± 120.2	1856.48 ± 101.5
Rutin	157.16 ± 9.6	58.48 ± 4.8	1824.05 ± 108.9	1652.05 ± 94.7
Luteolin	10.43 ± 1.2	8.43 ± 0.7	2.13 ± 0.2	2.88 ± 0.2
Quercitrin	5.71 ± 0.5	6.02 ± 0.5	21.90 ± 1.8	22.62 ± 2.1
Quercetin 3-β-D-glucoside	23.04 ± 2.2	73.07 ± 6.2	175.14 ± 11.6	183.11 ± 12.9
Quercetin	1.10 ± 0.1	10.69 ± 0.9	2.48 ± 0.1	17.09 ± 1.4
Kaempferol	10.20 ± 0.9	19.38 ± 1.4	52.05 ± 4.2	58.35 ± 5.1
*Isorhamnetin*	1.29 ± 0.1	0.47 ± 0.04	6.52 ± 0.5	3.59 ± 0.3
Daidzein	2.47 ± 0.2	1.65 ± 0.1	-	-
Formononetin	4.13 ± 0.3	2.72 ± 0.2	0.55 ± 0.04	0.30 ± 0.02
Genistein	8.35 ± 0.6	3.76 ± 0.2	2.21 ± 0.1	0.63 ± 0.05
Naringenin	0.05 ± 0.01	0.12 ± 0.01	0.47 ± 0.03	0.68 ± 0.04
Biochanin A	0.25 ± 0.02	0.36 ± 0.02	0.61 ± 0.03	0.67 ± 0.03
Vitexin	7.00 ± 0.5	65.30 ± 4.9	96.58 ± 8.9	158.15 ± 11.8

Conc. ASE—concentrated extract obtained after ASE extraction; Conc. LE—concentrated extract obtained after laser extraction. Results are expressed as mean *±* SD (*n* = 3).

**Table 2:** Total flavonoid content and antioxidant activity of analyzed extracts.

Sample		Total Polyphenol Content, μg CA/mL	Total Flavonoid Content, μg RE/mL	DPPH	Fe(III) Reducing Power
				EC_50_, µg/mL	
*M. sativa*	Conc. ASE	4584.5 ± 36.4	355.4 ± 8.4	278.7 ± 2.5 *	42.3 ± 0.3 *
	Conc. LE	4623.6 ± 29.8	426.7 ± 11.2	105.2 ± 1.1 *	40.9 ± 0.2 *
*S. virgaurea*	Conc. ASE	7928.7 ± 45.2	2198.7 ± 15.6	381.3 ± 2.9 *	58.7 ± 0.3 *
	Conc. LE	11,669.8 ± 94.6	1982.6 ± 13.8	198.4 ± 1.6 *	56.9 ± 0.4 *
Ascorbic	acid			39.4 ± 0.1	125.0 ± 1.1

Conc. ASE—concentrated extract obtained after ASE extraction; Conc. LE—concentrated extract obtained after laser extraction; CA: chlorogenic acid; RE: rutin equivalent. Results are expressed as mean *±* SD (*n* = 3); * *p* < 0.05.

**Table 3:** α-amylase and α-glucosidase enzyme inhibition of analyzed extracts.

Sample		α-Amylase Inhibition	α-Glucosidase Inhibition
		IC_50_ (µg/mL)	
*M. sativa*	Conc. ASE	23.9 ± 1.2 *	24.2 ± 0.9 *
	Conc. LE	26.8 ± 1.1 *	25.7 ± 1.1 *
*S. virgaurea*	Conc. ASE	33.9 ± 2.4 *	9.3 ± 0.9 *
	Conc. LE	32.1 ± 1.9 *	8.7 ± 0.6 *
Acarbose		24.2 ± 1.6	66.5 ± 4.2
Rutin		18.2 ± 2.4	8.6 ± 0.7

Conc. ASE—concentrated extract obtained after ASE extraction; Conc. LE—concentrated extract obtained after laser extraction; * *p* < 0.05 the α-amylase and α-glucosidase inhibition activity compared with polyphenol and flavonoid contents.

## Data Availability

Data are contained within the article.
